# Crystal structure determination, Hirshfeld surface analysis and energy frameworks of 6-phenyl­sulfonyl-6*H*-thieno[3,2-*c*]carbazole

**DOI:** 10.1107/S2056989018007971

**Published:** 2018-06-05

**Authors:** U. Mohamooda Sumaya, E. Sankar, K. Arasambattu MohanaKrishnan, K. Biruntha, G. Usha

**Affiliations:** a Department of Physics, Bharathi Women’s College (A), Chennai-108, Tamilnadu, India; b Department of organic Chemistry, University of Madras, Chennai-25, Tamilnadu, India; c PG and Research Department of Physics, Queen Mary’s College (A), Chennai-4, Tamilnadu, India

**Keywords:** crystal structure, carbazole, Hirshfeld surface analysis, two-dimensional fingerprint plot, energy frameworks

## Abstract

In the title compound, C_20_H_13_NO_2_S_2_, the carbazole ring system forms a dihedral angle of 89.08 (1)° with the sulfonyl-substituted phenyl ring. Intra­molecular C—H⋯O hydrogen bonds involving the sulfone O atoms and the carbazole moiety result in two *S*(6) rings. In the crystal, mol­ecules are linked *via* pairs of C—H⋯O hydrogen bonds forming inversion dimers with an *R*_2_^2^(12) graph-set motif. Analysis of the Hirshfeld surfaces and two-dimensional fingerprint plots was used to explore the distribution of weak inter­molecular inter­actions in the crystal structure.

## Abstract 2

Intra­molecular C—H⋯O hydrogen bonds involving the sulfone O atoms and the carbazole moiety result in two *S*(6) rings. In the crystal, mol­ecules are linked *via* pairs of C—H⋯O hydrogen bonds forming inversion dimers with an 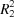
(12) graph-set motif.

## Chemical context   

Carbazole derivatives are among the most important and highly exploited heterocyclic compounds in the field of medicinal chemistry. They have been attractive to researchers because of their broad spectrum of biological activities, such as anti-oxidative (Tachibana *et al.*, 2001 ▸), anti­tumor (Itoigawa *et al.*, 2000 ▸), anti-inflammatory and anti­mutagenic (Ramsewak *et al.*, 1999 ▸), anti­biotic, anti­fungal and cytotoxic (Chakraborty *et al.*, 1965 ▸, 1978 ▸), pim kinase inhibitory (Giraud *et al.*, 2014 ▸), anti­microbial (Gu *et al.*, 2014 ▸) and anti-Alzheimer (Thiratmatrakul *et al.*, 2014 ▸). Carbazole derivatives are also used as precursor compounds for the synthesis of pyridocarbazole alkaloids (Karmakar *et al.*, 1991 ▸).
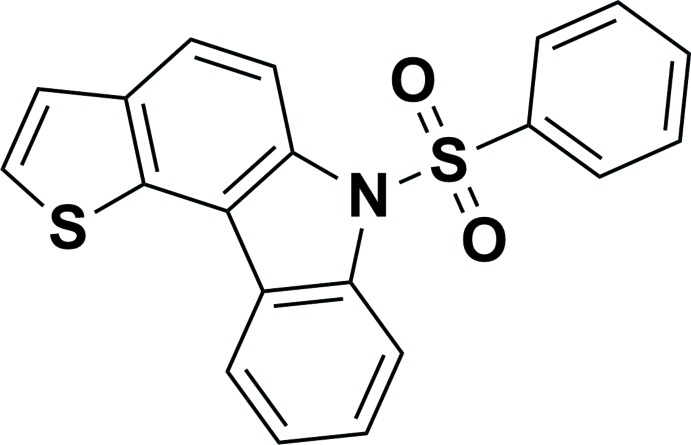


## Structural commentary   

**Figure 1:**
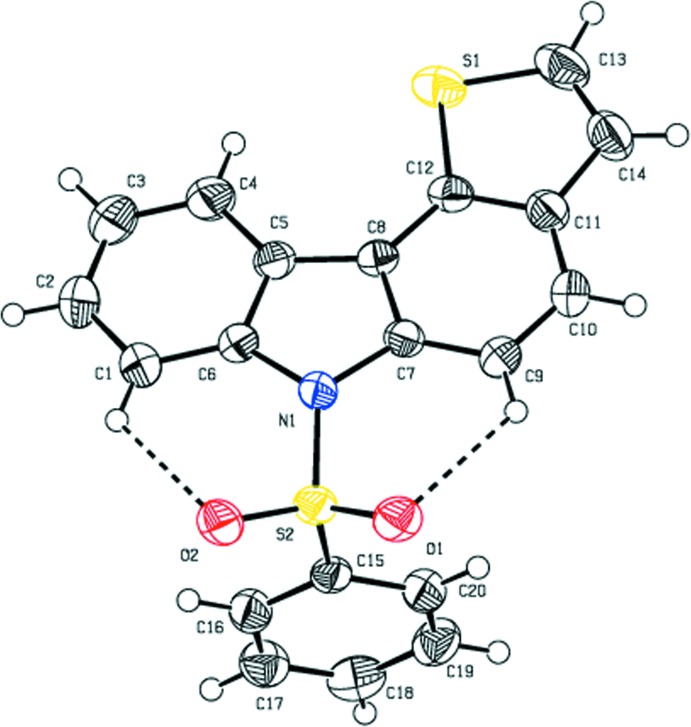
The mol­ecular structure of the title compound with the atom labelling. Displacement ellipsoids are drawn at the 50% probability level. Dashed lines indicate the intra­molecular C—H⋯O hydrogen bonds, which generate *S*(6) ring motifs.

The mol­ecular structure of the title compound is illustrated in Fig.1. The title compound comprises a carbazole ring system, which is attached to a phenyl sulfonyl ring and a thio­pene ring. The carbazole ring system forms a dihedral angle of 89.08 (1)° with the sulfonyl-substituted phenyl ring. The tetra­hedral configuration is distorted around the atom S2. The increase in the O2—S2—O1 angle [120.14 (9)°], with a simultaneous decrease in the N1—S2—C15 angle [104.96 (9)°] from the ideal tetra­hedral value (109.5°) are attributed to the Thorpe–Ingold effect (Bassindale, 1984 ▸). The N1—C6 [1.428 (2) Å] and N1—C7 [1.429 (2) Å] bond lengths in the mol­ecule are longer than the mean N*sp* ^2^—C*sp* ^2^ bond length value of 1.355 (14) Å (Allen *et al.*, 1987 ▸; Groom *et al.*, 2016 ▸). The elongation observed may be due to the electron-withdrawing character of the phenyl­sulfonyl group. The mol­ecular structure is stabilized by C1—H1⋯O2 and C9—H9⋯O1 intra­molecular inter­actions involving the sulfone oxygen atoms, which generate two *S*(6) ring motifs (Fig. 1 ▸).

## Supra­molecular features   

**Figure 2:**
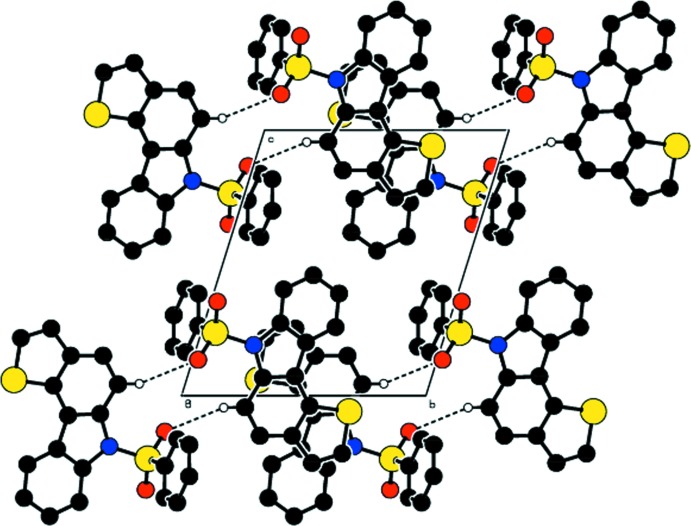
The crystal packing of the title compound, viewed along the *a* axis. Dashed lines indicate inter­molecular hydrogen bonds. For clarity, only the H atoms involved in these inter­actions have been included.

In the crystal packing (Fig. 2 ▸), the mol­ecules are linked *via* pairs of C—H⋯O hydrogen bonds (Table 1 ▸), forming inversion dimers with an 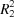
(12) graph-set motif. Each molecule is involved in the formation of two dimers that propagate as a ribbon in the *c*-axis direction.

## Hirshfeld surface analysis, inter­action energies and energy frameworks   

**Figure 3:**
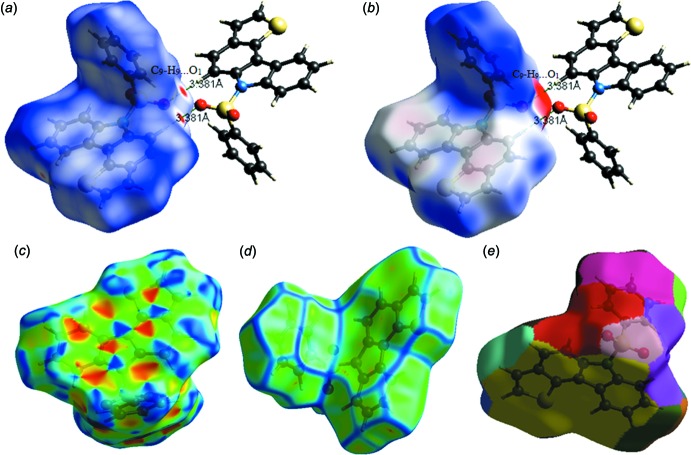
Hirshfeld surfaces for visualizing the inter­molecular contacts of the title compound: (*a*) *d*_norm_ highlighting the regions of C—H⋯O hydrogen bonds, (*b*) electrostatic potential, (*c*) shape index, (*d*) curvedness and (*e*) fragment patches.

In order to investigate the weak inter­molecular inter­actions in the crystal, the Hirshfeld surfaces (*d*_norm_, curvedness and shape index) and 2D fingerprint plots were generated using *CrystalExplorer 17.5* (Turner *et al.*, 2017 ▸). The *d*_norm_ mapping uses the normalized functions of *d*_i_ and *d*_e_ (Fig. 3 ▸ *a*), with white, red and blue coloured surfaces where *d*_i_ (*x* axis) and *d*_e_ (*y* axis) are the closest inter­nal and external distances from a given point on the Hirshfeld surface to the nearest atom. The white surface indicates those contacts with distances equal to the sum of van der Waals (vdW) radii, red indicates shorter contacts (< vdW radii) and blue longer contacts (> vdW radii). The electrostatic potential was also mapped on the Hirshfeld surface using a STO-3G basis set and the Hartee–Fock level of theory (Spackman *et al.*, 2008 ▸; Jayatilaka *et al.*, 2005 ▸). The C—H⋯O hydrogen-bond donors and acceptors are shown as blue and red regions around the atoms corresponding to positive and negative electrostatic potentials, respectively (Fig. 3 ▸ *b*). The presence of π–π stacking inter­actions is indicated by red and blue triangles on the shape-index surface (Fig. 3 ▸ *c*). Areas on the Hirshfeld surface with high curvedness tend to divide the surface into contact patches with each neighbouring mol­ecule. The coordination number in the crystal is defined by the curvedness of the Hirshfeld surface (Fig. 3 ▸ *d*). The nearest neighbour coordination environment of a mol­ecule is identified from the colour patches on the Hirshfeld surface depending on their closeness to adjacent mol­ecules (Fig. 3 ▸ *e*).

**Figure 4:**
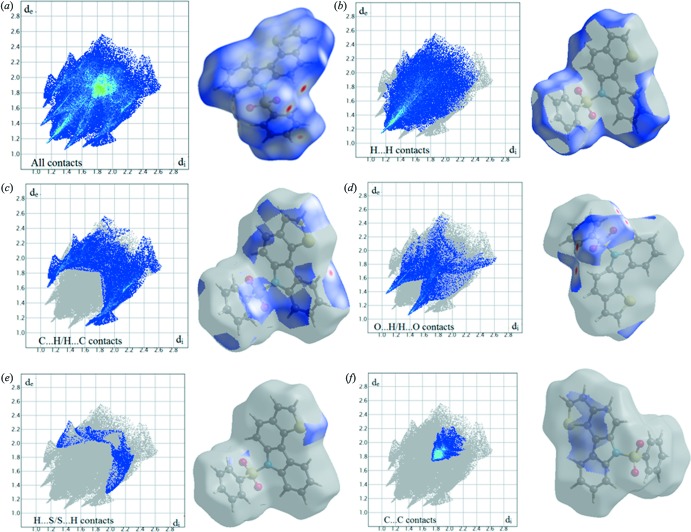
Two-dimensional fingerprint plots for the title compound showing the contributions of different types of inter­actions: (*a*) all inter­molecular contacts, (*b*) H⋯H contacts, (*c*) C⋯H/H⋯C contacts, (*d*) O⋯H/H⋯O contacts, (*e*) H⋯S/S⋯H contacts and (*f*) C⋯C contacts. The outline of the the full fingerprint is shown in gray. Surfaces to the right highlight the relevant surface patches associated with the specific contact type and are coloured as *d*_norm_.

Two-dimensional fingerprint plots showing the occurrence of all inter­molecular contacts (McKinnon *et al.*, 2007 ▸) are presented in Fig. 4 ▸ *a*. The fingerprint plot of H⋯H contacts, which represent the largest contribution to the Hirshfeld surfaces (40%), shows a distinct pattern with a minimum value of *d*_e_ = *d*_i_ ≃ 1.2 Å (Fig. 4 ▸ *b*). The C⋯H/H⋯C inter­actions appear as the next largest region of the fingerprint plot, highly concentrated at the edges, having almost the same *d*_e_ + *d*_i_ ≃ 2.7 Å (Fig. 4 ▸ *c*), with an overall Hirshfeld surface contribution of 24.1%. The O⋯H/H⋯O inter­actions on the fingerprint plot, which contribute 15.1% of the total Hirshfeld surface with *d*_e_ + *d*_i_ ≃ 2.5 Å (Fig. 4 ▸ *d*), are shown as two symmetrical narrow pointed wings. The H⋯S/S⋯H inter­actions cover only 3.5% (Fig. 4 ▸ *e*) of the surface. The C⋯C contacts, which are the measure of π–π stacking inter­actions, occupy 8.7% of the Hirshfeld surface and appear as a unique triangle at *d*_e_ = *d*_i_ ≃ 1.8 Å (Fig. 4 ▸ *f*). These are the weak inter­actions that contribute the most to the packing of the title compound.

**Figure 5:**
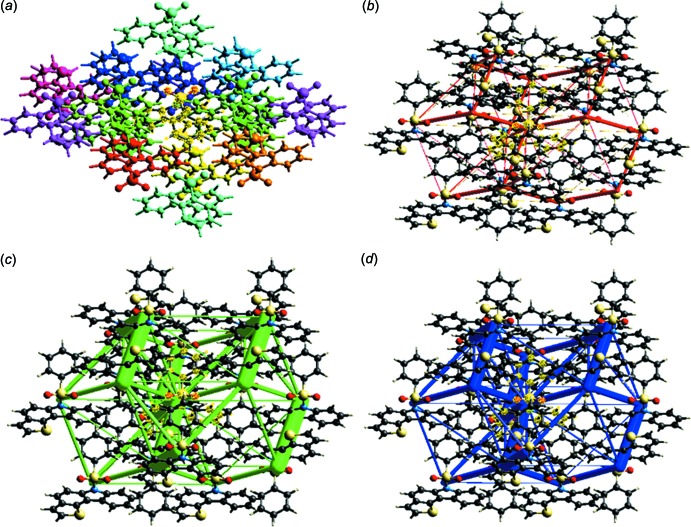
(*a*) Inter­actions between the selected reference mol­ecule (highlighted in yellow) and the mol­ecules present in a 3.8 Å cluster around it, (*b*) Coulomb energy framework, (*c*) dispersion energy framework and (*d*) total energy framework.

The inter­action energy between the mol­ecules is expressed in terms of four components: electrostatic, polarization, dispersion and exchange repulsion. These energies were obtained using monomer wavefunctions calculated at the B3LYP/6-31G(d,p) level. The total inter­action energy, which is the sum of scaled components, was calculated for a 3.8 Å radius cluster of mol­ecules around the selected mol­ecule (Fig. 5 ▸ *a*). The scale factors used in the CE-B3LYP benchmarked energy model (Mackenzie *et al.*, 2017 ▸) are given in Table 2 ▸. The inter­action energies calculated by the energy model reveal that the inter­actions in crystal have a significant contribution from dispersion components (Table 3 ▸). Using energy frameworks, the magnitudes of the inter­molecular inter­action energies are represented graphically and the supra­molecular architecture of the crystal structure is visualized. Energies between mol­ecular pairs are represented as cylinders joining the centroids of pairs of mol­ecules, with the cylinder radius proportional to the magnitude of the inter­action energy. Frameworks were constructed for *E*_elec_ as red cylinders, *E*_dis_ as green and *E*_tot_ as blue (Fig. 5 ▸ *b*–5*d*) and these cylinders represent the relative strength of mol­ecular packing in different directions.

## Synthesis and crystallization   

**Figure 6:**
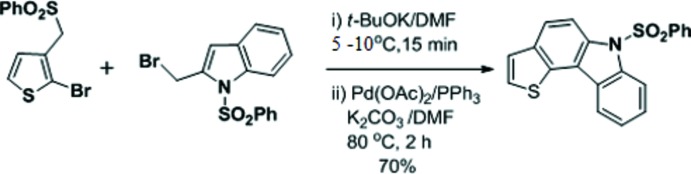
Reaction scheme.

The first step was the alkyl­ation of 2-bromo-3-(phenyl­sulfonyl­meth­yl)thio­phene (0.7 g, 2.21 mmol) with 2-bromo­methyl-1-phenyl­sulfonyl­indol (0.85 g, 2.43 mmol) using *t*-BuOK (0.37 g, 3.32 mmol) in DMF (20 mL) at 278–283 K for 15 min. After completion of the reaction, the reaction mixture was poured into crushed ice. The solid obtained was filtered and dried to afford the alkyl­ated sulfone (1.16 g) as a colourless solid. To a solution of the crude alkyl­ated sulfone (1.16 g, 1.97 mmol) in DMF (15 mL), Pd(OAc)_2_ (0.04 g, 0.19 mmol), PPh_3_ (0.10 g, 0.39 mmol) and K_2_CO_3_ (0.55 g, 3.94 mmol) were added. Then the reaction mixture was heated at 353 K for 2 h. After that, the reaction mixture was filtered through a celite bed and washed with ethyl acetate (2 × 10 mL). The combined organic layer was washed with water (3 × 20 mL) and dried (Na_2_SO_4_). Removal of the solvent followed by column chromatographic purification (silica gel, 100% hexa­ne) afforded 6-(phenyl­sulfon­yl)-6*H*-thieno[3,2-*c*]carbazole (0.50 g, 70%) as a colourless solid (Fig. 6 ▸). Diffraction-quality crystals were obtained from the product by slow evaporation using chloro­form as a solvent; m.p. 417–419 K.

## Refinement   

Crystal data, data collection and structure refinement details are summarized in Table 4 ▸. All H atoms were positioned geometrically (C—H = 0.93 Å) and refined using a riding model with *U*_iso_(H) = 1.2*U*_eq_(C). In the final refinement, reflection (001), which was obstructed by the beam stop, was omitted.

## Supplementary Material

Crystal structure: contains datablock(s) I. DOI: 10.1107/S2056989018007971/fy2128sup1.cif

Structure factors: contains datablock(s) I. DOI: 10.1107/S2056989018007971/fy2128Isup2.hkl

Supporting information file. DOI: 10.1107/S2056989018007971/fy2128Isup3.cml

CCDC reference: 1825258

Additional supporting information:  crystallographic information; 3D view; checkCIF report

## Figures and Tables

**Table 1:** Hydrogen-bond geometry (Å, °) *Cg*5 is the centroid of the C15–C20 ring.

*D*—H⋯*A*	*D*—H	H⋯*A*	*D*⋯*A*	*D*—H⋯*A*
C1—H1⋯O2	0.93	2.34	2.935 (3)	121
C1—H1⋯O2^i^	0.93	2.62	3.443 (3)	148
C9—H9⋯O1	0.93	2.35	2.949 (3)	122
C9—H9⋯O1^ii^	0.93	2.57	3.382 (2)	146
C13—H13⋯*Cg*5^iii^	0.93	2.82	3.604 (2)	143

Symmetry codes: (i) 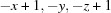; (ii) 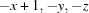; (iii) 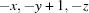.

**Table 2:** Scale factors for benchmarked energy model

Energy model	*k*_elec_	*k*_pol_	*k*_energy-dispersive_	*k*_rep_
CE-B3LYP⋯B3LYP/6–31G(d,p) electron densities	1.057	0.740	0.871	0.618

**Table 3:** Inter­action energies (kJ mol^−1^) between a reference mol­ecule and its neighbours *N* is the number of equivalent neighbours, *R* is the distance between mol­ecular centroids (mean atomic position) in Å. The colours identify mol­ecules in Fig. 5 ▸ *a*, with the reference mol­ecule shown in grey.

Colour	*N*	symmetry	*R*	*E*_elec_	*E*_pol_	*E*_energy-dispersive_	*E*_rep_	*E*_total_
Red	1	inversion	9.29	−3.7	−1.5	−27.5	14.6	−20.0
Orange	1	inversion	8.65	0.9	−1.4	−23.3	10.3	−14.0
Yellow	1	inversion	6.18	−12.2	−2.6	−83.1	54.3	−53.7
Green	2	translation	12.53	1.7	−0.5	−7.3	2.4	−3.4
Lime	2	translation	9.88	−2.4	−0.6	−19.5	14.0	−11.3
Aqua	2	translation	7.65	−4.5	−2.1	−12.1	5.4	−13.5
Cyan	1	inversion	7.79	−17.5	−4.8	−23.5	16.7	−32.2
Blue	1	inversion	8.76	−19.3	−5.0	−26.9	22.3	−33.8
Indigo	1	inversion	5.84	−11.7	−2.7	−87.7	51.5	−58.9
Purple	2	translation	11.22	1.9	−0.4	−6.9	3.6	−2.0
Pink	1	inversion	10.79	−2.6	−0.4	−8.1	2.1	−8.8

**Table 4:** Experimental details

Crystal data	
Chemical formula	C_20_H_13_NO_2_S_2_
*M*_r_	363.43
Crystal system, space group	Triclinic, *P* 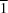
Temperature (K)	298
*a*, *b*, *c* (Å)	7.6461 (8), 9.8772 (9), 11.2191 (12)
α, β, γ (°)	72.571 (5), 88.496 (6), 86.144 (6)
*V* (Å^3^)	806.54 (14)
*Z*	2
Radiation type	Mo *K*α
μ (mm^−1^)	0.34
Crystal size (mm)	0.25 × 0.20 × 0.20

Data collection	
Diffractometer	Bruker Kappa APEXII CCD
Absorption correction	Multi-scan (*SADABS*; Bruker, 2012 ▸)
*T*_min_, *T*_max_	0.921, 0.934
No. of measured, independent and observed [*I* > 2σ(*I*)] reflections	16616, 3174, 2458
*R*_int_	0.036
(sin θ/λ)_max_ (Å^−1^)	0.617

Refinement	
*R*[*F* ^2^ > 2σ(*F* ^2^)], *wR*(*F* ^2^), *S*	0.034, 0.091, 1.03
No. of reflections	3102
No. of parameters	226
H-atom treatment	H-atom parameters constrained
Δρ_max_, Δρ_min_ (e Å^−3^)	0.24, −0.41

Computer programs: *APEX2*, *SAINT* and *XPREP* (Bruker, 2012 ▸), *SHELXS97* (Sheldrick, 2008 ▸), *SHELXL2014* (Sheldrick, 2015 ▸) and *ORTEP-3 for Windows* (Farrugia, 2012 ▸).

## supplementary crystallographic information

### Crystal data

**Table:**

C_20_H_13_NO_2_S_2_	*Z* = 2
*M**_r_* = 363.43	*F*(000) = 376
Triclinic, *P*1	*D*_x_ = 1.497 Mg m^−^^3^
*a* = 7.6461 (8) Å	Mo *K*α radiation, λ = 0.71073 Å
*b* = 9.8772 (9) Å	Cell parameters from 3175 reflections
*c* = 11.2191 (12) Å	θ = 2.4–28.8°
α = 72.571 (5)°	µ = 0.34 mm^−^^1^
β = 88.496 (6)°	*T* = 298 K
γ = 86.144 (6)°	Needle, colourless
*V* = 806.54 (14) Å^3^	0.25 × 0.20 × 0.20 mm

### Data collection

**Table:**

Bruker Kappa APEXII CCD diffractometer	2458 reflections with *I* > 2σ(*I*)
Radiation source: fine-focus sealed tube	*R*_int_ = 0.036
ω and φ scan	θ_max_ = 26.0°, θ_min_ = 2.4°
Absorption correction: multi-scan (SADABS; Bruker, 2012)	*h* = −9→9
*T*_min_ = 0.921, *T*_max_ = 0.934	*k* = −12→12
16616 measured reflections	*l* = −13→13
3174 independent reflections	

### Refinement

**Table:**

Refinement on *F*^2^	0 restraints
Least-squares matrix: full	Hydrogen site location: inferred from neighbouring sites
*R*[*F*^2^ > 2σ(*F*^2^)] = 0.034	H-atom parameters constrained
*wR*(*F*^2^) = 0.091	*w* = 1/[σ^2^(*F*_o_^2^) + (0.0389*P*)^2^ + 0.3787*P*] where *P* = (*F*_o_^2^ + 2*F*_c_^2^)/3
*S* = 1.03	(Δ/σ)_max_ < 0.001
3102 reflections	Δρ_max_ = 0.24 e Å^−^^3^
226 parameters	Δρ_min_ = −0.41 e Å^−^^3^

### Special details

**Table:**

Geometry. All esds (except the esd in the dihedral angle between two l.s. planes) are estimated using the full covariance matrix. The cell esds are taken into account individually in the estimation of esds in distances, angles and torsion angles; correlations between esds in cell parameters are only used when they are defined by crystal symmetry. An approximate (isotropic) treatment of cell esds is used for estimating esds involving l.s. planes.

### Fractional atomic coordinates and isotropic or equivalent isotropic displacement parameters (Å^2^)

**Table:**

	*x*	*y*	*z*	*U*_iso_*/*U*_eq_	
C1	0.3947 (3)	0.2820 (2)	0.4004 (2)	0.0462 (5)	
H1	0.4480	0.1960	0.4478	0.055*	
C2	0.3350 (4)	0.3854 (3)	0.4540 (2)	0.0590 (7)	
H2	0.3496	0.3685	0.5394	0.071*	
C3	0.2541 (4)	0.5137 (3)	0.3848 (2)	0.0614 (7)	
H3	0.2148	0.5806	0.4242	0.074*	
C4	0.2317 (3)	0.5426 (2)	0.2583 (2)	0.0478 (6)	
H4	0.1776	0.6287	0.2118	0.057*	
C5	0.2909 (3)	0.44110 (19)	0.20083 (18)	0.0327 (4)	
C6	0.3716 (3)	0.31203 (19)	0.27287 (18)	0.0331 (4)	
C7	0.3683 (2)	0.30993 (19)	0.06791 (17)	0.0313 (4)	
C8	0.2892 (2)	0.43910 (19)	0.07345 (17)	0.0300 (4)	
C9	0.3877 (3)	0.2747 (2)	−0.04358 (19)	0.0395 (5)	
H9	0.4417	0.1880	−0.0450	0.047*	
C10	0.3247 (3)	0.3722 (2)	−0.1510 (2)	0.0432 (5)	
H10	0.3350	0.3499	−0.2259	0.052*	
C11	0.2454 (3)	0.5042 (2)	−0.15089 (19)	0.0378 (5)	
C12	0.2289 (2)	0.53705 (19)	−0.03801 (19)	0.0335 (4)	
C13	0.1126 (3)	0.7312 (2)	−0.2176 (2)	0.0526 (6)	
H13	0.0633	0.8143	−0.2728	0.063*	
C14	0.1762 (3)	0.6197 (2)	−0.2532 (2)	0.0499 (6)	
H14	0.1756	0.6172	−0.3354	0.060*	
C15	0.2718 (3)	−0.02095 (18)	0.26426 (18)	0.0318 (4)	
C16	0.2018 (3)	−0.0612 (2)	0.38416 (19)	0.0392 (5)	
H16	0.2624	−0.0493	0.4508	0.047*	
C17	0.0401 (3)	−0.1193 (2)	0.4027 (2)	0.0458 (5)	
H17	−0.0082	−0.1480	0.4826	0.055*	
C18	−0.0494 (3)	−0.1347 (2)	0.3035 (2)	0.0482 (6)	
H18	−0.1589	−0.1727	0.3166	0.058*	
C19	0.0212 (3)	−0.0946 (2)	0.1843 (2)	0.0473 (5)	
H19	−0.0408	−0.1052	0.1178	0.057*	
C20	0.1835 (3)	−0.0388 (2)	0.1641 (2)	0.0405 (5)	
H20	0.2332	−0.0135	0.0845	0.049*	
O1	0.55980 (19)	0.02188 (14)	0.13545 (14)	0.0426 (4)	
O2	0.56255 (19)	0.02622 (14)	0.35412 (13)	0.0423 (4)	
S1	0.13085 (7)	0.70590 (5)	−0.05968 (6)	0.04557 (17)	
S2	0.47505 (6)	0.05704 (5)	0.23770 (5)	0.03323 (14)	
N1	0.4252 (2)	0.23071 (16)	0.19079 (14)	0.0332 (4)	

### Atomic displacement parameters (Å^2^)

**Table:**

	*U*^11^	*U*^22^	*U*^33^	*U*^12^	*U*^13^	*U*^23^
C1	0.0662 (15)	0.0380 (12)	0.0332 (12)	−0.0017 (10)	−0.0041 (11)	−0.0091 (9)
C2	0.094 (2)	0.0533 (14)	0.0340 (12)	−0.0060 (13)	0.0035 (13)	−0.0189 (11)
C3	0.095 (2)	0.0454 (14)	0.0500 (15)	−0.0002 (13)	0.0092 (14)	−0.0256 (12)
C4	0.0623 (15)	0.0344 (11)	0.0468 (13)	0.0051 (10)	0.0037 (11)	−0.0143 (10)
C5	0.0353 (11)	0.0284 (9)	0.0352 (11)	−0.0037 (8)	0.0016 (8)	−0.0105 (8)
C6	0.0376 (11)	0.0299 (10)	0.0331 (10)	−0.0034 (8)	0.0006 (8)	−0.0111 (8)
C7	0.0332 (10)	0.0278 (9)	0.0313 (10)	−0.0025 (7)	−0.0018 (8)	−0.0060 (8)
C8	0.0289 (10)	0.0267 (9)	0.0345 (10)	−0.0039 (7)	0.0000 (8)	−0.0091 (8)
C9	0.0520 (13)	0.0321 (10)	0.0361 (11)	0.0008 (9)	0.0007 (10)	−0.0138 (9)
C10	0.0570 (14)	0.0434 (12)	0.0312 (11)	−0.0052 (10)	−0.0007 (10)	−0.0138 (9)
C11	0.0395 (12)	0.0363 (11)	0.0349 (11)	−0.0065 (9)	−0.0049 (9)	−0.0053 (9)
C12	0.0315 (10)	0.0274 (9)	0.0394 (11)	−0.0043 (8)	−0.0022 (8)	−0.0057 (8)
C13	0.0534 (15)	0.0415 (13)	0.0508 (14)	0.0009 (10)	−0.0151 (11)	0.0051 (11)
C14	0.0554 (15)	0.0509 (14)	0.0369 (12)	−0.0075 (11)	−0.0106 (11)	−0.0012 (10)
C15	0.0354 (11)	0.0243 (9)	0.0354 (11)	0.0050 (7)	−0.0050 (8)	−0.0095 (8)
C16	0.0436 (12)	0.0368 (11)	0.0365 (11)	0.0035 (9)	−0.0044 (9)	−0.0111 (9)
C17	0.0472 (13)	0.0432 (12)	0.0455 (13)	−0.0025 (10)	0.0070 (11)	−0.0116 (10)
C18	0.0366 (12)	0.0391 (12)	0.0694 (16)	−0.0011 (9)	0.0007 (11)	−0.0173 (11)
C19	0.0470 (13)	0.0443 (12)	0.0546 (14)	−0.0018 (10)	−0.0144 (11)	−0.0199 (11)
C20	0.0470 (13)	0.0366 (11)	0.0373 (11)	0.0013 (9)	−0.0045 (10)	−0.0106 (9)
O1	0.0446 (9)	0.0362 (8)	0.0472 (9)	0.0064 (6)	0.0048 (7)	−0.0150 (6)
O2	0.0417 (8)	0.0387 (8)	0.0427 (9)	0.0048 (6)	−0.0145 (7)	−0.0066 (6)
S1	0.0462 (3)	0.0317 (3)	0.0526 (4)	0.0048 (2)	−0.0053 (3)	−0.0043 (2)
S2	0.0342 (3)	0.0271 (2)	0.0365 (3)	0.00447 (18)	−0.0042 (2)	−0.0077 (2)
N1	0.0429 (10)	0.0271 (8)	0.0287 (8)	0.0026 (7)	−0.0039 (7)	−0.0077 (7)

### Geometric parameters (Å, º)

**Table:**

C1—C2	1.380 (3)	C11—C14	1.437 (3)
C1—C6	1.385 (3)	C12—S1	1.7323 (19)
C1—H1	0.9300	C13—C14	1.338 (3)
C2—C3	1.386 (4)	C13—S1	1.722 (3)
C2—H2	0.9300	C13—H13	0.9300
C3—C4	1.374 (3)	C14—H14	0.9300
C3—H3	0.9300	C15—C20	1.387 (3)
C4—C5	1.392 (3)	C15—C16	1.387 (3)
C4—H4	0.9300	C15—S2	1.760 (2)
C5—C6	1.401 (3)	C16—C17	1.382 (3)
C5—C8	1.435 (3)	C16—H16	0.9300
C6—N1	1.428 (2)	C17—C18	1.374 (3)
C7—C8	1.392 (3)	C17—H17	0.9300
C7—C9	1.397 (3)	C18—C19	1.382 (3)
C7—N1	1.429 (2)	C18—H18	0.9300
C8—C12	1.399 (3)	C19—C20	1.378 (3)
C9—C10	1.373 (3)	C19—H19	0.9300
C9—H9	0.9300	C20—H20	0.9300
C10—C11	1.401 (3)	O1—S2	1.4233 (15)
C10—H10	0.9300	O2—S2	1.4236 (15)
C11—C12	1.399 (3)	S2—N1	1.6572 (16)
			
C2—C1—C6	117.0 (2)	C8—C12—S1	128.08 (16)
C2—C1—H1	121.5	C14—C13—S1	113.43 (17)
C6—C1—H1	121.5	C14—C13—H13	123.3
C1—C2—C3	122.1 (2)	S1—C13—H13	123.3
C1—C2—H2	118.9	C13—C14—C11	112.8 (2)
C3—C2—H2	118.9	C13—C14—H14	123.6
C4—C3—C2	120.5 (2)	C11—C14—H14	123.6
C4—C3—H3	119.8	C20—C15—C16	121.34 (19)
C2—C3—H3	119.8	C20—C15—S2	119.11 (16)
C3—C4—C5	119.1 (2)	C16—C15—S2	119.55 (16)
C3—C4—H4	120.5	C17—C16—C15	118.7 (2)
C5—C4—H4	120.5	C17—C16—H16	120.6
C4—C5—C6	119.44 (19)	C15—C16—H16	120.6
C4—C5—C8	132.60 (19)	C18—C17—C16	120.2 (2)
C6—C5—C8	107.96 (16)	C18—C17—H17	119.9
C1—C6—C5	121.89 (18)	C16—C17—H17	119.9
C1—C6—N1	130.16 (18)	C17—C18—C19	120.8 (2)
C5—C6—N1	107.91 (17)	C17—C18—H18	119.6
C8—C7—C9	122.62 (18)	C19—C18—H18	119.6
C8—C7—N1	108.02 (16)	C20—C19—C18	120.0 (2)
C9—C7—N1	129.33 (17)	C20—C19—H19	120.0
C7—C8—C12	118.03 (17)	C18—C19—H19	120.0
C7—C8—C5	108.38 (16)	C19—C20—C15	119.0 (2)
C12—C8—C5	133.58 (18)	C19—C20—H20	120.5
C10—C9—C7	117.95 (19)	C15—C20—H20	120.5
C10—C9—H9	121.0	C13—S1—C12	91.09 (11)
C7—C9—H9	121.0	O1—S2—O2	120.14 (9)
C9—C10—C11	121.76 (19)	O1—S2—N1	106.94 (8)
C9—C10—H10	119.1	O2—S2—N1	106.82 (8)
C11—C10—H10	119.1	O1—S2—C15	108.46 (9)
C12—C11—C10	119.05 (18)	O2—S2—C15	108.51 (9)
C12—C11—C14	111.32 (19)	N1—S2—C15	104.96 (9)
C10—C11—C14	129.6 (2)	C6—N1—C7	107.66 (15)
C11—C12—C8	120.57 (18)	C6—N1—S2	124.02 (13)
C11—C12—S1	111.34 (15)	C7—N1—S2	124.80 (13)
			
C6—C1—C2—C3	0.5 (4)	C12—C11—C14—C13	0.1 (3)
C1—C2—C3—C4	−0.5 (4)	C10—C11—C14—C13	−178.9 (2)
C2—C3—C4—C5	0.2 (4)	C20—C15—C16—C17	−0.4 (3)
C3—C4—C5—C6	0.1 (3)	S2—C15—C16—C17	178.53 (15)
C3—C4—C5—C8	−179.2 (2)	C15—C16—C17—C18	−0.8 (3)
C2—C1—C6—C5	−0.2 (3)	C16—C17—C18—C19	0.9 (3)
C2—C1—C6—N1	176.9 (2)	C17—C18—C19—C20	0.2 (3)
C4—C5—C6—C1	−0.1 (3)	C18—C19—C20—C15	−1.4 (3)
C8—C5—C6—C1	179.39 (19)	C16—C15—C20—C19	1.6 (3)
C4—C5—C6—N1	−177.79 (18)	S2—C15—C20—C19	−177.40 (15)
C8—C5—C6—N1	1.7 (2)	C14—C13—S1—C12	0.00 (19)
C9—C7—C8—C12	−0.6 (3)	C11—C12—S1—C13	0.04 (16)
N1—C7—C8—C12	177.62 (16)	C8—C12—S1—C13	179.58 (19)
C9—C7—C8—C5	−179.87 (18)	C20—C15—S2—O1	−30.91 (17)
N1—C7—C8—C5	−1.6 (2)	C16—C15—S2—O1	150.12 (15)
C4—C5—C8—C7	179.3 (2)	C20—C15—S2—O2	−162.97 (15)
C6—C5—C8—C7	0.0 (2)	C16—C15—S2—O2	18.06 (18)
C4—C5—C8—C12	0.2 (4)	C20—C15—S2—N1	83.12 (16)
C6—C5—C8—C12	−179.1 (2)	C16—C15—S2—N1	−95.86 (16)
C8—C7—C9—C10	−0.4 (3)	C1—C6—N1—C7	179.9 (2)
N1—C7—C9—C10	−178.23 (19)	C5—C6—N1—C7	−2.7 (2)
C7—C9—C10—C11	0.9 (3)	C1—C6—N1—S2	20.2 (3)
C9—C10—C11—C12	−0.5 (3)	C5—C6—N1—S2	−162.32 (14)
C9—C10—C11—C14	178.4 (2)	C8—C7—N1—C6	2.7 (2)
C10—C11—C12—C8	−0.6 (3)	C9—C7—N1—C6	−179.27 (19)
C14—C11—C12—C8	−179.65 (18)	C8—C7—N1—S2	162.11 (14)
C10—C11—C12—S1	179.00 (16)	C9—C7—N1—S2	−19.8 (3)
C14—C11—C12—S1	−0.1 (2)	O1—S2—N1—C6	−166.71 (15)
C7—C8—C12—C11	1.1 (3)	O2—S2—N1—C6	−36.89 (18)
C5—C8—C12—C11	−179.87 (19)	C15—S2—N1—C6	78.21 (17)
C7—C8—C12—S1	−178.41 (14)	O1—S2—N1—C7	37.08 (18)
C5—C8—C12—S1	0.6 (3)	O2—S2—N1—C7	166.91 (15)
S1—C13—C14—C11	0.0 (3)	C15—S2—N1—C7	−78.00 (17)

### Hydrogen-bond geometry (Å, º)

Cg5 is the centroid of the C15–C20 ring.

**Table:**

*D*—H···*A*	*D*—H	H···*A*	*D*···*A*	*D*—H···*A*
C1—H1···O2	0.93	2.34	2.935 (3)	121
C1—H1···O2^i^	0.93	2.62	3.443 (3)	148
C9—H9···O1	0.93	2.35	2.949 (3)	122
C9—H9···O1^ii^	0.93	2.57	3.382 (2)	146
C13—H13···*Cg*5^iii^	0.93	2.82	3.604 (2)	143

Symmetry codes: (i) −*x*+1, −*y*, −*z*+1; (ii) −*x*+1, −*y*, −*z*; (iii) −*x*, −*y*+1, −*z*.
